# Low-cost yield-driven design of antenna structures using response-variability essential directions and parameter space reduction

**DOI:** 10.1038/s41598-022-19411-1

**Published:** 2022-09-07

**Authors:** Anna Pietrenko-Dabrowska, Slawomir Koziel, Lukasz Golunski

**Affiliations:** 1 Faculty of Electronics, Telecommunications and Informatics, Gdansk University of Technology, 80-233 Gdańsk, Poland; 2 Engineering Optimization & Modeling Center, Reykjavik University, 102 Reykjavík, Iceland

**Keywords:** Electrical and electronic engineering, Computational science

## Abstract

Quantifying the effects of fabrication tolerances and uncertainties of other types is fundamental to improve antenna design immunity to limited accuracy of manufacturing procedures and technological spread of material parameters. This is of paramount importance especially for antenna design in the industrial context. Degradation of electrical and field properties due to geometry parameter deviations often manifests itself as, e.g., center frequency shifts or compromised impedance matching. Improving antenna performance at the presence of uncertainties is typically realized through maximization of the fabrication yield. This is normally carried out at the accuracy level of full-wave electromagnetic (EM) analysis, which incurs considerable computational expenses. The involvement of surrogate modeling techniques is the most common approach to alleviating these difficulties, yet conventional modeling methods suffer to a great extent form the curse of dimensionality. This work proposes a technique for low-cost yield optimization of antenna structures. It capitalizes on meticulous definition of the domain of the metamodel constructed for statistical analysis purposes. The domain is spanned by a limited number of essential directions being the most influential in terms of affecting antenna responses in the frequency bands of interest. These directions are determined through an automated decision-making process based on the assessment of the circuit response variability. Our approach permits maintaining small domain volume, which translates into low cost of surrogate model setup, while providing sufficient room for yield improvement. The presented method is validated using three antenna structures and favorably compared to several surrogate-assisted benchmark methods. EM-driven Monte Carlo simulation is also conducted to verify reliability of the yield optimization process.

## Introduction

Antenna structures are typically developed and optimized in the nominal sense, i.e., by assuming that the physical prototypes retain the geometry and material parameter values determined in the design process. This neglects possible fabrication tolerances but also other types of uncertainties, related to the lack of precise knowledge of material parameters (e.g., substrate permittivity), or operating conditions (input power levels, temperature, mechanical bending). Needless to say, the effects of tolerances are always detrimental to antenna characteristics, and, in many cases, may lead to violating the prescribed design specifications. Consequently, uncertainty quantification is of high practical importance, for both academia and industry. Among the two groups of uncertainties, the most commonly considered in antenna design are the aleatory ones^1^. These are primarily deviations of antenna geometry parameters from their nominal values, resulting from limited accuracy of the manufacturing and/or assembly procedures, and described by probability distributions^2,3^. The latter are process-specific but also dependent on interrelations between antenna dimensions. To a certain extent, the undesirable effects of tolerances may be reduced through stochastic design that aim at maximizing suitably selected statistical figures of merit, e.g., the yield^4^. The second major class of uncertainties, epistemic (or systematic) ones^5,6^, associated with the limited knowledge of operating conditions, are typically handled—at the design stage—by ensuring sufficient performance of the antenna system within the required ranges of the conditions (e.g., bending radius of a wearable antenna).

Uncertainty quantification (UQ) of high-frequency structures including antennas is an intricate task because of the associated computational costs. Reliable estimation of the statistical moments of antenna responses or other figures (e.g., the yield), requires a large number of electromagnetic (EM) simulations, especially when using conventional approaches, such as EM-driven Monte Carlo analysis^7^. A reduction of the CPU expenses can be achieved by simplified formulations, e.g., worst case analysis^8^, which, however, does not give an accurate account for the effects of tolerances. Nowadays, the most commonly used approach to computationally-efficient statistical analysis constitute surrogate modeling methods, both data-driven (e.g., response surface approximation^9^, neural networks^10^, polynomial chaos expansion, PCE^11^^,^^12^), but also physics-based (e.g., space mapping^13,14^). The bottleneck of surrogate-based UQ is the construction of the surrogate model itself, which becomes problematic for structures described by larger numbers of geometry parameters. In practice, due to high nonlinearity of antenna characteristics, this might be difficult even beyond a few (e.g., four to six) parameters. Among available mitigation methods, the following are worth mentioning: dimensionality reduction (e.g., by employing principal component analysis^15^), variable-resolution approaches (e.g., co-kriging^16^), hybrid methods (e.g. PC kriging that employs a PCE surrogate instead of a low-order polynomial as a trend function^17^), or model order reduction^18^.

As mentioned before, reducing the undesirable effects of manufacturing tolerances can be accomplished at the antenna development stage by appropriate adjustment of its geometry parameters. This process is referred to as robust or tolerance-aware design^19–21^, and aims at improving a selected statistical figure of merit. Needless to say, with antenna designs becoming increasingly more sophisticated, e.g., due to incorporation of metamaterials (e.g., in the form of metasurfaces)^40–43^, robust design is more important than ever before. If the specifications are of minimax-type (e.g., expressed through acceptable levels of antenna responses over target bandwidths^22^), the merit function is typically the yield, maximized under the assumed probability distributions for parameter deviations. An alternative would be to maximize the input tolerance levels for which the design specifications can still be met (e.g., tolerance hypervolume maximization^23^). In either case, robust design a CPU-intensive endeavor due to the necessity of solving multiple UQ sub-problems as a part of the process^24^. Understandably, straightforward EM-driven tolerance-aware optimization is computationally prohibitive in most cases. Instead, surrogate-assisted methods are commonly employed, which are probably the only practical options^25–29^. Similarly as for statistical analysis, the surrogate modeling methods utilized in the context of robust design include response surface approximations, neural networks^30^, variable-fidelity methods (space mapping^31^), as well as PCE^32^. On the other hand, a construction of surrogates for stochastic optimization purposes is more challenging: due to design relocation, the model domain has to cover broader ranges of antenna parameters. This can be alleviated by means of sequential approximate optimization (SAO)^33^, where the metamodel is only built in a proximity of the current design, and its domain is shifted down the optimization path. This way, the cost of setting up each (localized) surrogate is considerably lower at the expense of having the model constructed several times in the optimization process. The response feature approach^34^ is a yet another possibility. It capitalizes on weakly-nonlinear dependence between geometry parameters and the coordinates of appropriately selected characteristic points of the system responses, which are used instead of the complete outputs to evaluate the statistical figures of merit. The mentioned relationships permit a construction of accurate models using small training data sets^35,36^, thereby improving the computational efficiency of the search process.

This work introduces an alternative technique for expedited yield-driven parameter tuning of antenna structures. The presented methodology employs kriging metamodels established in the domain spanned by the selected directions within the parameter space, corresponding to the most significant variations of antenna responses. These directions form an orthonormal basis, and are identified through an automated decision-making process that involves auxiliary optimization sub-problems. Within this approach, the domain of the surrogate has a small volume (therefore, the model can be set up at low computational cost), yet, due to employing problem-specific knowledge, it exhibits a sufficient extent along the relevant directions. The latter enables one-step optimization without relocating the domain as in SAO-based methods. Our framework has been validated using four microstrip antennas, a single-, dual-, and triple-band ones. The average computational cost is only 115 EM analyses of the respective structure, with the reliability verified with the use of simulation-driven Monte Carlo simulation. At the same time, the presented technique has been shown to offer significant savings in comparison to benchmark surrogate-assisted procedures.

## Yield-driven design using response-variability essential directions

Here, we introduce the proposed approach to computationally-efficient tolerance-aware of antenna structures. We begin by formulating the yield optimization task; for the sake of clarification, we focus on the case of multi-band antennas (section “Yield optimization task. Problem formulation for multi-band antenna case”). Section “Benchmark algorithms” discusses surrogate-assisted uncertainty quantification and outlines two generic algorithms later utilized as the benchmark techniques. The methodology presented in this work is elaborated on in sections “Robust design using essential directions” and “Complete yield optimization algorithm”, where the concept and identification procedure of essential directions is explained, and the entire yield optimization process is summarized, respectively.

### Yield optimization task. Problem formulation for multi-band antenna case

A formulation of the robust design task depends on a specific choice of the statistical figures of merit, the type of antenna response, and the type of design specifications assumed for the structure. Here, we discuss multi-band antennas with design requirements defined in a minimax sense for their input characteristics. Let *f*_0*k*_, *k* = 1, …, *N*, be the target operating frequencies, and *B*_*k*_ be the target bandwidths of the *N*-band antenna. Also, let *S*_11_(***x***,*f*) denote the antenna reflection response at the design ***x*** (a vector of geometry parameters) and frequency *f*. The design specifications are satisfied if

1 $$ \max \left\{ {f \in \bigcup\nolimits_{k = 1}^{N} {\left[ {f_{0k} - B_{k} /2,f_{0k} + B_{k} /2} \right]} \;:\;|S_{11} ({\mathbf{x}},f)|} \right\} \le S_{\max } $$

where *S*_max_ is the threshold value, typically − 10 dB. Satisfaction of (1) is equivalent to the antenna matching being no worse than *S*_max_ within all target operating bands.

The nominal design, denoted as ***x***^(0)^, is obtained by minimizing the in-band reflection in the sense of (1). More specifically, we have

2 $$ {\mathbf{x}}^{(0)} = \arg \mathop {\min }\limits_{{\mathbf{x}}} \left\{ {\max \left\{ {f \in \bigcup\nolimits_{k = 1}^{N} {\left[ {f_{0k} - B_{k} /2,f_{0k} + B_{k} /2} \right]} {:}\;\; |S_{11} ({\mathbf{x}},f)|} \right\}} \right\} $$

The vector ***x***^(0)^ is obtained without taking into account any uncertainties. It corresponds to the design that provides the best possible matching under the assumptions that the actual antenna dimensions (upon prototype fabrication) are identical with those obtained in the optimization process.

In practice, the actual parameter values ***x*** are different from the ‘ideal’ ones due to manufacturing tolerances. The statistical variations of ***x*** with respect to ***x***^(0)^ are described by means of the assumed probability density function *p*(***x***,***x***^(0)^). The latter may be joint Gaussian with zero mean and a variance *σ*, or uniform with a maximum deviation *d*_max_. The distributions may be independent, or correlated (quantified using a specific covariance matrix), depending on parameter interrelations. For example, the deviation for the spacing between coupled transmission lines is strictly correlated with the deviation of their widths, etc.

In this work, we use yield^13^ as the statistical performance metric. The yield *Y* is the likelihood of satisfying the design specifications given the density function *p*(). It is defined as

3 $$ Y\left( {{\mathbf{x}}^{(0)} } \right) = \int\limits_{{X_{f} }} {p\left( {{\mathbf{x}},{\mathbf{x}}^{(0)} } \right)d{\mathbf{x}}} $$

where *X*_*f*_ denotes the feasible space, here, the set of parameter vectors for which the condition (1) holds.

Because *X*_*f*_ is not known explicitly, the formula (3) is typically evaluated through stochastic integration, e.g., using Monte Carlo (MC) analysis. In MC, *Y* is estimated as

4 $$ Y\left( {{\mathbf{x}}^{(0)} } \right) = p^{ - 1} \sum\nolimits_{k = 1}^{p} {H\left( {{\mathbf{x}}^{(k)} } \right)} $$

where ***x***^(*k*)^, *k* = 1, …, *N*_*r*_, are random observables drawn according to the density function *p*(.). The function *H*(***x***) is defined as *H*(***x***) = 1 if the condition (1) is satisfied, and *H*(***x***) = 0 otherwise.

The robust design task can be then formulated as yield maximization, with the optimum design obtained by solving

5 $$ {\mathbf{x}}^{*} = \arg \mathop {\min }\limits_{{\mathbf{x}}} \{ - Y({\mathbf{x}})\} $$

The starting point for (5) is typically the nominal design ***x***^(0)^.

Needless to say, maximizing yield is of utmost practical importance, particularly from the industrial perspective: reducing the number of fabricated devices that do not satisfy the prescribed specs directly translates into reliability of the systems the device is part of, and, consequently, the overall manufacturing costs and the profits.

### Benchmark algorithms

Executing (4), let alone (5), directly at the level of full-wave electromagnetic model of the antenna structure at hand, is computationally inefficient or even prohibitive. The practical way of carrying out EM-driven uncertainty quantification is the employment of surrogate modeling techniques. The main idea and specific techniques have been mentioned in section “Introduction”. In this section, we consider two simple methods representing different approaches to surrogate-assisted robust design, which will be used as benchmark algorithms in section “Numerical verification”. Both algorithms are characterized in Table 1.

**Table 1:** Generic surrogate-assisted yield optimization algorithms (benchmark methods in section “Numerical verification”).

Algorithm	1	2
Optimization type	One-shot search	Sequential approximation optimization
Surrogate model	Kriging interpolation	Kriging interpolation
Solution method	Directly solve x* = argmin{***x*** ∈ *X*_*S*_ : –*Y*(***x***)} within the model domain; *Y*(***x***) estimated using the surrogate	Obtain approximations ***x***^(*i*)^, *i* = 0, 1, …, of x* as ***x***^(*i*+1)^ = argmin{***x*** ∈ *X*_*S.i*_: –*Y*_*s*_^(*i*)^(***x***)}, where whereas *Y*_*s*_^(*i*)^ is the yield estimated using the *i*th surrogate model
Model domain	*X*_*S*_ = [***x***^(0)^−***d***, ***x***^(0)^ + ***d***], with ***d*** = [*d*_1_ … *d*_*n*_]^*T*^, *d*_*k*_ = 10*d*_max_, *k* = 1, …, *n*	*X*_*S.i*_ = [***x***^(*i*)^−***d***, ***x***^(*i*)^ + ***d***], with ***d*** = [*d*_1_ … *d*_*n*_]^*T*^, *d*_*k*_ = 3*d*_max_, *k* = 1, …, *n*
Termination condition	Algorithm finished upon finding x*	Convergence in argument ||***x***^(*i*)^−***x***^(*i*+1)^||< *ε* (user-defined)
Pros	Simple implementation	Lower cost of surrogate construction (smaller domain)
Cons	Potentially high cost of constructing the surrogate in a larger domain	Iterative process requiring domain relocation and rendition of multiple surrogates

^#^*d*_max_ is the maximum deviation in the case of uniform distribution, or 3σ for Gaussian distribution of variance σ.

Algorithm 1 is a one-shot technique, with the metamodel built in a relatively large vicinity of the nominal design, so that the robust optimum can be found by optimizing the metamodel once, without the necessity of further improvements. This is a simple scheme, yet the initial cost of training data acquisition may be large, typically a few hundred for data points for medium-dimensionality antenna structures. Algorithm 2 adopts a sequential approximate optimization scheme, where the surrogate is constructed in a smaller vicinity of the current design, which lowers the cost of training data acquisition. In each iteration, the surrogate is rendered in the domain centered at the current solution, and the process is continued upon convergence. Typically, Algorithm 2 exhibits better efficacy over Algorithm 2 at the expense of more complex implementation.

### Robust design using essential directions

The major concept proposed in this work is to combine the advantages of one-shot and sequential approximate optimization approaches in order to reduce the cost of yield maximization process while ensuring its reliability. Toward this end, we aim at setting up a single surrogate model, the domain of which extends sufficiently in essential (referred to as essential) directions, while being restricted in the remaining ones. This section introduces the notion essential directions, the procedure for their identification, as well as provides a definition of the surrogate model domain involving thereof.

#### Essential directions. Knowledge-based identification procedure

The essential directions are established to maximize the antenna response variability in the frequency ranges of interest. In particular, if the design specifications are formulated as in (1) for the reflection coefficient *S*_11_(***x***,*f*), the relevant frequency range *F* = [*f*_0.1_−*B*_1_/2, *f*_0.1_ + *B*_1_/2] ∪ … ∪ [*f*_0.*N*_−*B*_*N*_/2, *f*_0.*N*_ + *B*_*N*_/2] (the symbol ∪ stands for the set-theory sum). The response change between designs ***x***_1_ and ***x***_2_ can be quantified as

6 $$ D_{v} \left( {{\mathbf{x}}_{1} ,{\mathbf{x}}_{2} } \right) = \sqrt {\int\limits_{F} {\left[ {|S_{11} ({\mathbf{x}}_{1} ,f)| - |S_{11} ({\mathbf{x}}_{2} ,f)|} \right]^{2} df} } $$

Note that (6) is nothing but the *L*-square norm computed over the frequency range of interest.

Our objective is to find an orthonormal basic of vectors {***v***^(*j*)^}_*j*_ = 1, …, *n*, that correspond to the maximum values of *D*_*v*_, within the entire parameter space for vector ***v***^(1)^, the subspace being an orthogonal complement of the subspace spanned by ***v***^(1)^ for vector ***v***^(2)^, and so on. Clearly, maximization of the variability metric *D*_*v*_ cannot be realized directly as the level of EM simulation model due to excessive computational costs. Here, we use a first-order Taylor model of antenna responses instead. Let ***G***(***x***,*f*) be the gradient of *S*_11_(***x***,*f*). The first-order model is defined as

7 $$   S_{{11 \cdot L}} ({\mathbf{x}},f) = S_{{11}} \left( {{\mathbf{x}}^{{(0)}} } \right) + {\mathbf{G}}\left( {{\mathbf{x}}^{{(0)}} ,f} \right) \cdot \left( {{\mathbf{x}} - {\mathbf{x}}^{{(0)}} } \right)  $$

Using (7), we defined the approximated variability metric *D*_*v.L*_ as

8 $$ D_{v.L} \left( {{\mathbf{x}}_{1} ,{\mathbf{x}}_{2} } \right) = \sqrt {\int\limits_{F} {\left[ {|S_{11.L} ({\mathbf{x}}_{1} ,f)| - |S_{11} ({\mathbf{x}}_{2} ,f)|} \right]^{2} df} } $$

The vector ***v***^(1)^ is then obtained as

9 $$ {\mathbf{v}}^{(1)} = \arg \mathop {\max }\limits_{{{\mathbf{v}};\;||{\mathbf{v}}|| = 1}} D_{v.L} \left( {{\mathbf{x}}^{(0)} + {\mathbf{v}},{\mathbf{x}}^{(0)} } \right) $$

The remaining directions ***v***^(2)^, ***v***^(3)^, …, are obtained using a similar process. Nevertheless, in order to ensure that {***v***^(*j*)^} forms an orthonormal basis, the vector ***v***^(*j*+1)^ is found in the orthogonal complement of the subspace spanned by {***v***^(1)^, …, ***v***^(*j*)^}. More specifically, we have

10 $$ {\mathbf{v}}^{(j + 1)} = \arg \mathop {\max }\limits_{{{\overline{\mathbf{v}}}}} D_{v.L} \left( {{\mathbf{x}}^{(0)} + {\overline{\mathbf{v}}},{\mathbf{x}}^{(0)} } \right) $$

where ${\overline{\mathbf{v}}}$ are of the form

11 $$ {\overline{\mathbf{v}}} = \frac{{P^{(j)} ({\mathbf{v}})}}{{||P^{(j)} ({\mathbf{v}})||}} $$

with

12 $$ P^{(j)} ({\mathbf{v}}) = {\mathbf{v}} - \sum\nolimits_{k = 1}^{j} {{\mathbf{v}}^{(k)} \left[ {({\mathbf{v}}^{(k)} )^{T} {\mathbf{v}}} \right]} $$

The condition (11) ensures that all considered vectors are of unity length, whereas condition (12) allows to restrict the search to vectors that are orthogonal to ***v***^(*k*)^, *k* = 1, …, *j*.

**Figure 1:**
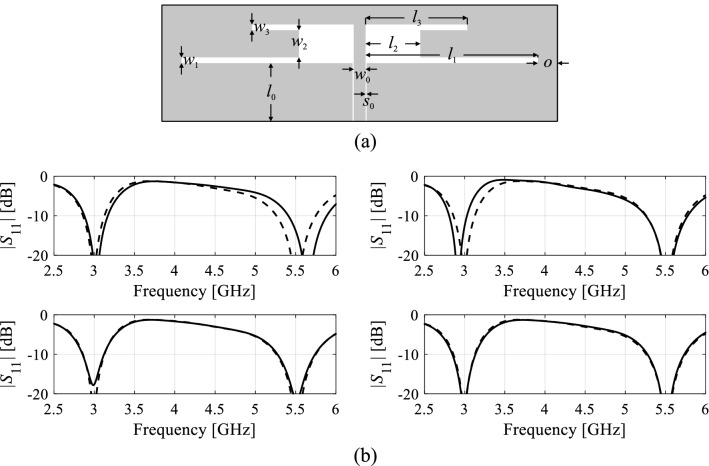
Dual-band dipole antenna and its response variability: (**a**) antenna geometry, (**b**) EM-evaluated reflection characteristics |*S*_11_(***x***,*f*)| (- - -) and |*S*_11_(***x*** + *h****v***^(*j*)^,*f*)| (—) for *j* = 1, 2, 3, and 4 (from top-left to right-bottom). Here, *h* = 0.03 to obtain visually noticeable response changes. Note that the first two directions contribute to the vast majority of response alterations.

Figure 1 shows response variability for one of the microstrip antennas considered in section “Numerical verification”. The values of *D*_*v*_ (cf. (6)) along the vectors ***v***^(1)^ through ***v***^(4)^ (normalized to the highest one) obtained using the Taylor model (cf. (8)) are 1.00, 0.62, 0.21, and 0.07, whereas EM-simulated ones are 1.00, 0.76, 0.26, and 0.08, which indicates good predictive power of the linear model. At the same time, it can be noted that the first two essential directions are responsible for the majority of antenna response changes, therefore, it is reasonable to define the domain of the surrogate model mainly along these directions.

In practice, the number *N*_*s*_ of directions selected for defining the domain can be determined using the contribution coefficients *c*_*j*_ of the form

13 $$ c_{j} = \frac{{\sqrt {\sum\nolimits_{k = 1}^{j} {\left[ {D_{v.L} ({\mathbf{x}}^{(0)} + {\mathbf{v}}^{(k)} ,{\mathbf{x}}^{(0)} )} \right]}^{2} } }}{{\sqrt {\sum\nolimits_{k = 1}^{n} {\left[ {D_{v.L} ({\mathbf{x}}^{(0)} + {\mathbf{v}}^{(k)} ,{\mathbf{x}}^{(0)} )} \right]}^{2} } }} $$

which quantify the contribution of the first *j* essential directions to the total antenna response change.

If the threshold value is set to *c*_min_ (e.g., 0.9), we can obtain *N*_*s*_ as

14 $$ N_{s} = \arg \mathop {\min }\limits_{{}} \left\{ {j \in \{ 1,2,...,n\} :c_{j} \ge c_{\min } } \right\} $$

For the example considered in Fig. 1, we get *N*_*s*_ = 2 for *c*_min_ = 0.9. As a matter of fact, we have *c*_2_ = 0.98. In most practical cases, we have *N*_*s*_ = 2 or 3.

#### Surrogate model domain definition

The surrogate model domain *X*_*S*_ is an *n*-dimensional interval in the parameter space, spanned by all vectors ***v***^(*j*)^, *j* = 1, …, *n*. However, its extent is larger along the first *N*_*s*_ essential directions (cf. (14)), whereas it is smaller along the remaining ones. We define

15 $$ X_{S} = \left\{ \begin{gathered} {\mathbf{x}}^{(0)} + \sum\limits_{j = 1}^{n} {a_{j} d_{j} {\mathbf{v}}^{(j)} } \\ - 1 \le a_{j} \le 1,\;j = 1,...,n \\ \end{gathered} \right\} $$

In other words, *X*_*S*_ is the set of all points of the form ***x***^(0)^ + ∑_*j* = 1,…,*n*_* a*_*j*_*d*_*j*_***v***^(*j*)^ with *a*_*j*_ being between –1 and 1, and *d*_*j*_ being the size factors. We use the following setup

16 $$ d_{j} = \left\{ \begin{gathered} Md_{\max } \sqrt n \;\;\;\;\;j = 1,...,N_{s} \hfill \\ d_{\max } \sqrt n \;\;\;\;\;\;j = N_{s} + 1,...,n \hfill \\ \end{gathered} \right. $$

where *M* is set to 5 for the numerical experiments of section “Numerical verification” to ensure sufficient room for yield improvement along the first *N*_*s*_ essential directions. Recall that *d*_max_ is the maximum assumed parameter deviation. The factor *n*^1/2^ is to ensure that for any point ***x*** that is moved along the *N*_*s*_ directions (with respect to the nominal design ***x***^(0)^), the vicinity of ***x*** in the form of the interval [− *d*_max_,*d*_max_]^*n*^ is in *X*_*S*_. The latter is necessary to ensure that yield estimation can be realized within the surrogate model domain regardless of a particular allocation of vectors ***v***^(*j*)^.

Determining the surrogate model domain as in (15), (16), allows for maintaining its small size, while covering the directions of essential variations of antenna responses. On the one hand, this allows for establishing the surrogate using a small number of training data samples. On the other hand, it makes it possible to carry out yield optimization without the necessity of iterating the process, in particular, to rebuild the surrogate.

In this work, the surrogate model is defined in *X*_*S*_ using kriging interpolation^37^. Antenna optimization is conducted by solving (5) with the yield estimated using the surrogate, the same way as in Algorithm 1 (cf. Table 1).

### Complete yield optimization algorithm

**Figure 2:**
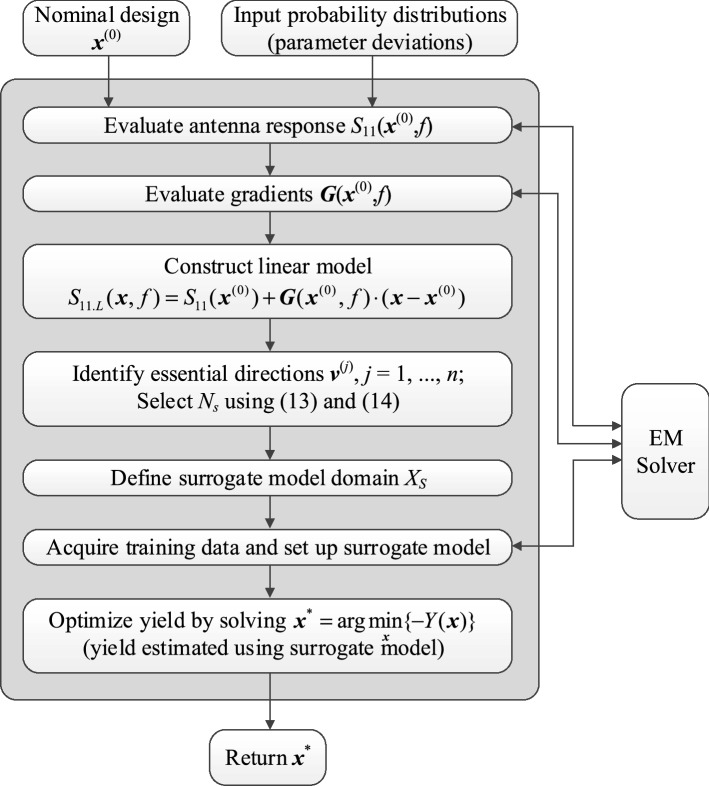
Flow diagram of the proposed surrogate-assisted robust design procedure involving essential directions.

The flow diagram of the proposed surrogate-assisted robust design procedure involving essential directions has been shown in Fig. 2. Given the nominal design and input probability distributions quantifying fabrication tolerances, the principal directions are first established as described in section “Robust design using essential directions”. The kriging surrogate model is then constructed in the domain *X*_*S*_ defined as in section “Surrogate model domain definition”. Yield optimization is carried out at the level of surrogate model by directly solving (5). The surrogate is set up to ensure its sufficient predictive power, which is achieved by maintaining the relative RMS error of about one percent.

## Numerical verification

The presented robust design technique is validated here using several microstrip antenna structures, including single-, dual- and triple-band ones. The results are compared to the benchmark algorithms outlined in section “Benchmark algorithms”. Moreover, the Monte Carlo analysis using full-wave simulations is applied to verify the reliability of estimating the yield with the use of the surrogate.

### Test cases

**Figure 3:**
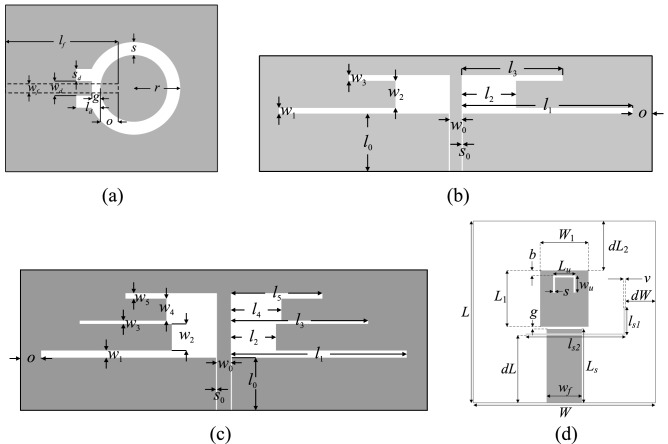
Verification case studies: (**a**) microstrip-fed ring slot antenna (Antenna I)^36^, (**b**) dual-band uniplanar dipole antenna (Antenna II)^38^, (**c**) triple-band uniplanar dipole antenna (Antenna III)^38^, (**c**) triple-band U-slotted patch antenna (Antenna IV)^39^.

The robust design procedure considered in this work is validated using four antenna structures shown in Fig. 3. These include a microstrip-fed ring slot antenna (Antenna I)^36^, Fig. 3a, a dual- (Antenna II^38^, Fig. 3b) and triple-band uniplanar dipole antenna (Antenna III^38^, Fig. 3c), as well as a triple-band U-slotted patch antenna with defected ground (Antenna IV^39^, Fig. 3d). The above antenna set has been specifically chosen because narrow- and multi-band structures are prone to considerable variations of their operating bandwidths due to fabrication tolerances (mostly in the form of frequency shifts). Also, simultaneous control of several independent operating bands is more challenging than handling a single band. From this perspective, using, e.g., a broadband antenna as a case study is of limited interest due to small expected changes of the fabrication yield.

Table 2 provides relevant information about all structures, including substrate data, geometry parameters, design specifications, as well as nominal designs, optimized to improve impedance matching of the respective antennas within their operating bands. The computational models of Antennas I through IV are implemented in CST Microwave Studio and simulated using the time-domain solver.

**Table 2:** Verification case studies.

	Case study			
	Antenna I	Antenna II	Antenna III	Antenna IV
Substrate	*ε*_*r*_ = 2.0, *h* = 0.76 mm	*ε*_*r*_ = 3.5, *h* = 0.76 mm	*ε*_*r*_ = 3.5, *h* = 0.76 mm	*ε*_*r*_ = 3.2, *h* = 3.1 mm
Design parameters	***x*** = [*l*_*f*_* l*_*d*_* w*_*d*_* r s s*_*d*_* o g*]^*T*^	***x*** = [*l*_1_ *l*_2_ *l*_3_ *w*_1_ *w*_2_ *w*_3_]^*T*^	***x*** = [*l*_1_ *l*_2_ *l*_3_ *l*_4_ *l*_5_ *w*_1_ *w*_2_ *w*_3_ *w*_4_ *w*_5_]^*T*^	***x*** = [*L*_1_ *L*_*s*_* L*_*u*_* W W*_1_ *dL dW g l*_*s*1_ *l*_*s*2_ *w*_*u*_]^*T*^
Other parameters	–	*l*_0_ = 30, *w*_0_ = 3, *s*_0_ = 0.15, and *o* = 5	*l*_0_ = 30, *w*_0_ = 3, *s*_0_ = 0.15, and *o* = 5	*b* = 1, *w*_*f*_ = 7.4, *s* = 0.5, *w* = 0.5, *dL*_2_ = *L*_1_; *L* = *L*_*s*_ + *g* + *L*_1_ + *dL*_2_
Operating bands	4.15 GHz to 4.85 GHz	8-percent fractional bandwidth w.r.t. center frequencies 3.0 GHz and 5.5 GHz	4-percent fractional bandwidth w.r.t. center frequencies 2.45 GHz, 3.6 GHz, and 5.3 GHz	120 MHz bandwidth centered at operating frequencies 3.5 GHz, 5.8 GHz, and 7.5 GHz
Nominal design	***x***^(0)^ = [20.28 6.54 0.24 11.83 2.95 6.77 7.85 2.23]^*T*^	***x***^(0)^ = [30.47 11.60 19.20 0.47 2.46 1.30]^*T*^	***x***^(0)^ = [35.42 11.54 26.07 8.09 17.14 0.60 0.99 1.44 0.78 1.17]^*T*^	***x***^(0)^ = [11.52 19.03 6.44 52.47 10.81 18.89 10.22 0.32 4.88 23.11 0.20]^*T*^

### Experimental setup and results

For all considered antenna structures, the manufacturing tolerances are assumed to follow independent uniform distributions of the maximum deviation of *d*_max_ = 0.05 mm. The proposed robust design approach has been executed with the surrogate model domain set up as in section “Surrogate model domain definition”, and using *N*_*s*_ = 2, i.e., extended towards the first two essential components.

The yield-optimized designs obtained for Antennas I through IV are the following:

- Antenna I: x^*^ = [20.32 6.56 0.17 11.86 2.95 6.75 7.86 2.25]^*T*^;
- Antenna II: x^*^ = [30.43 11.63 19.25 0.46 2.39 1.31]^*T*^;
- Antenna III: x^*^ = [35.36 11.58 26.03 8.11 17.16 0.60 0.96 1.41 0.81 1.21]^*T*^;
- Antenna IV: x^*^ = [11.43 19.13 6.43 52.36 10.90 18.90 10.25 0.27 4.90 23.13 2.00]^*T*^.

**Figure 4:**
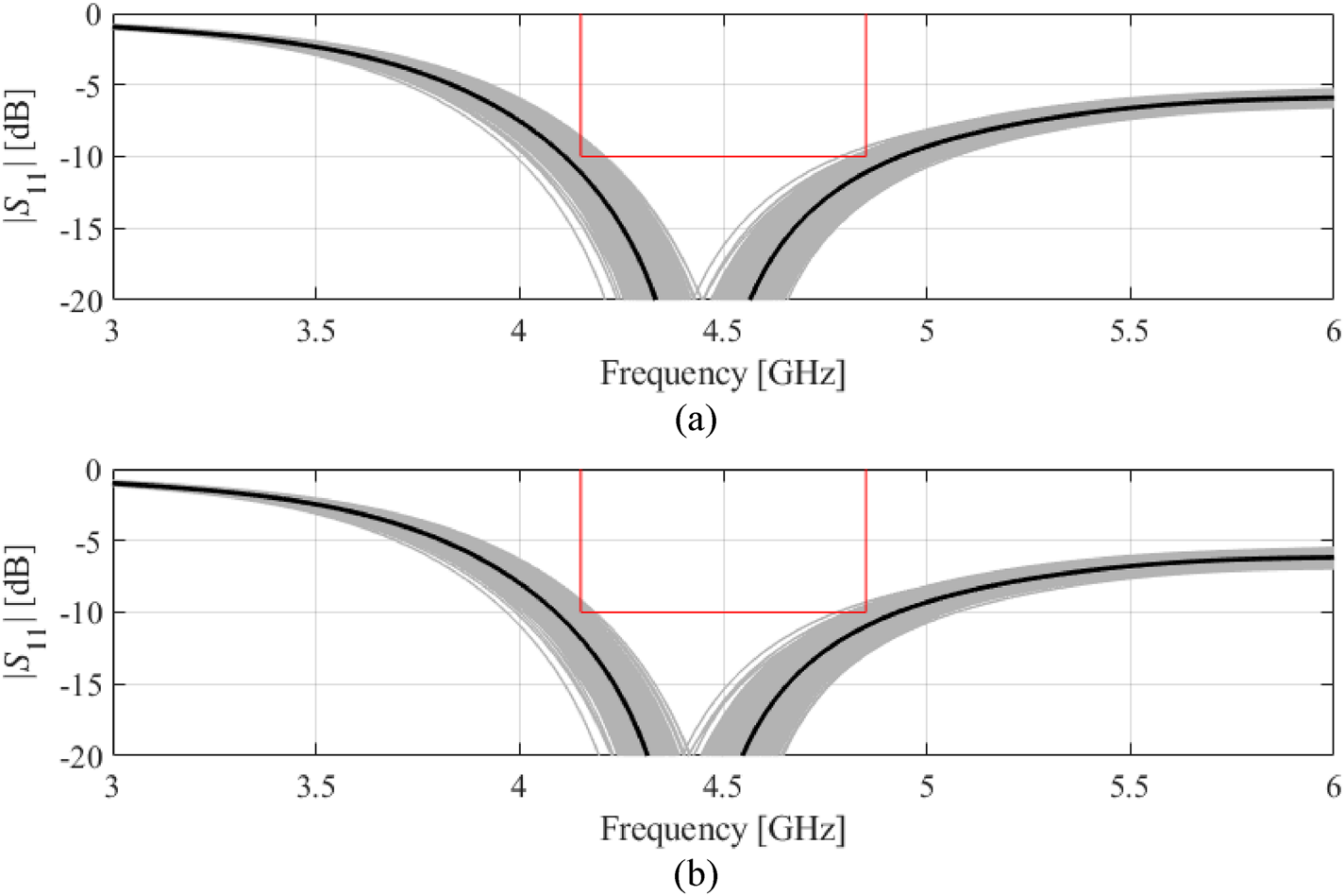
Visualization of EM-driven Monte Carlo simulation of Antenna I using 500 samples. Gray plots represent random observables, whereas the black plot is the antenna response at the center design (nominal for (**a**) and yield-optimized for (**b**)). Target operating bandwidth and reflection level has been marked using vertical and horizonal lines, respectively: (**a**) nominal design, (**b**) robust design.

**Figure 5:**
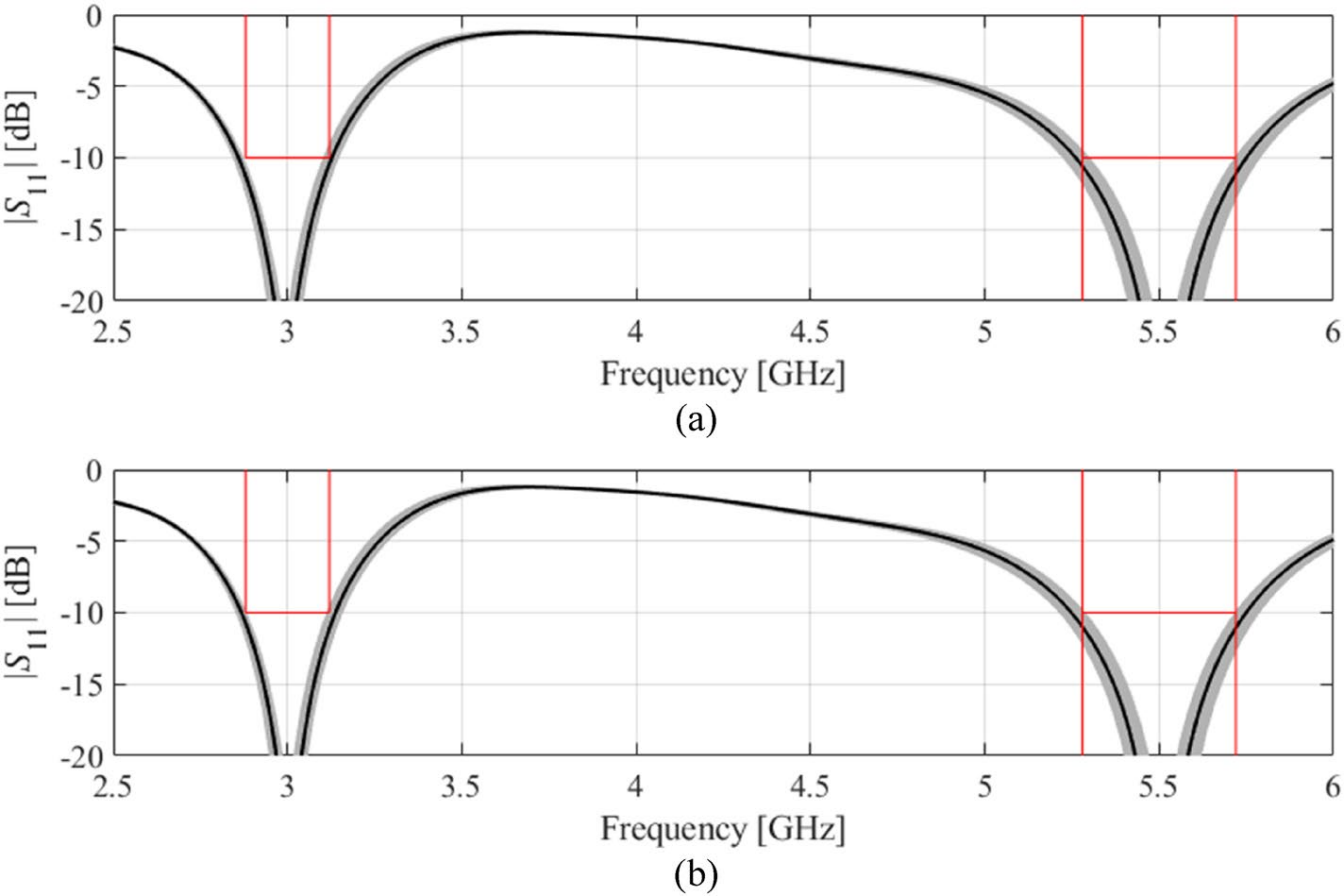
Visualization of EM-driven Monte Carlo simulation of Antenna II using 500 samples. Gray plots represent random observables, whereas the black plot is the antenna response at the center design (nominal for (**a**) and yield-optimized for (**b**)). Target operating bandwidths and reflection level has been marked using vertical and horizonal lines, respectively: (**a**) nominal design, (**b**) robust design.

**Figure 6:**
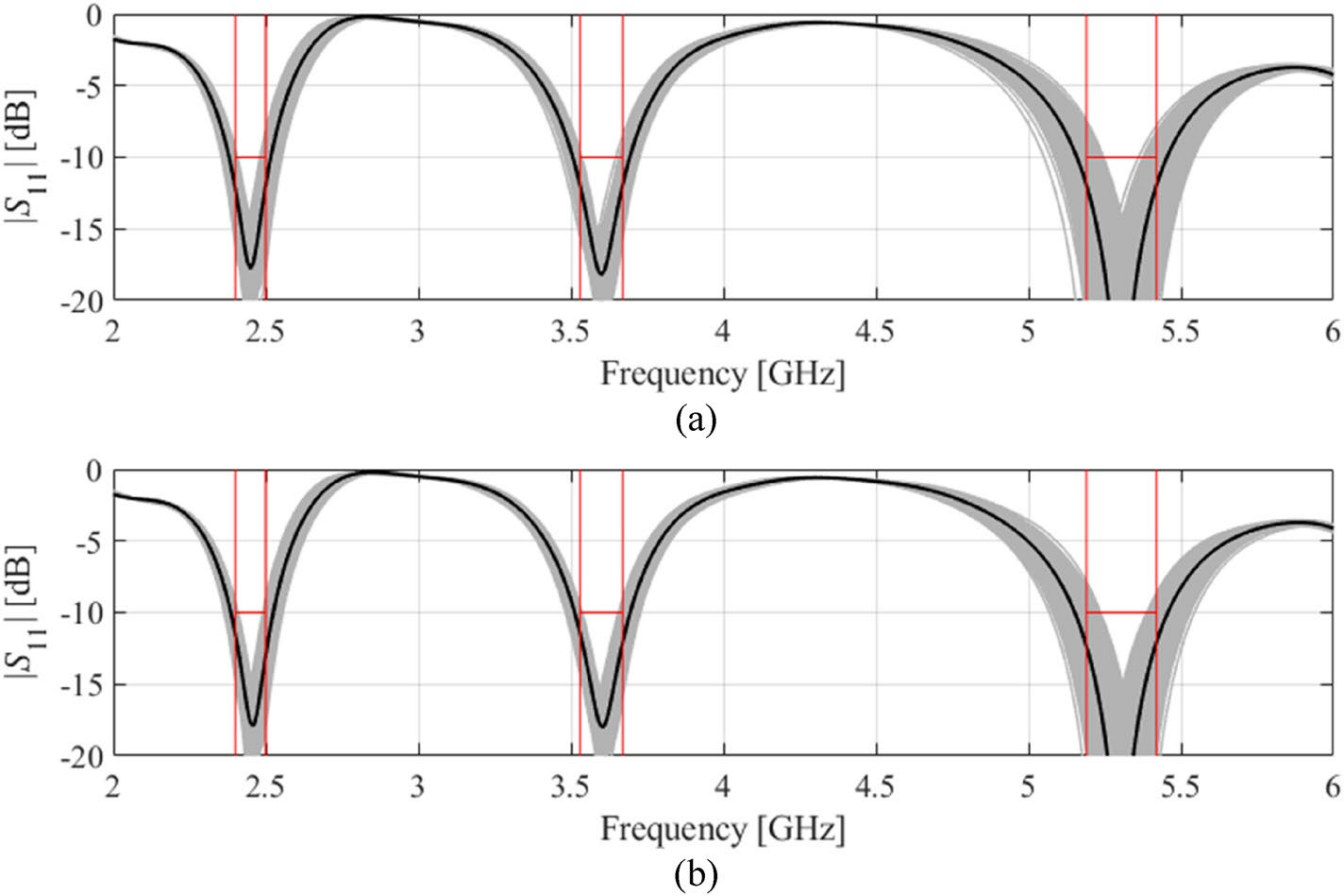
Visualization of EM-driven Monte Carlo simulation of Antenna III using 500 samples. Gray plots represent random observables, whereas the black plot is the antenna response at the center design (nominal for (**a**) and yield-optimized for (**b**)). Target operating bandwidths and reflection level has been marked using vertical and horizonal lines, respectively: (**a**) nominal design, (**b**) robust design.

**Figure 7:**
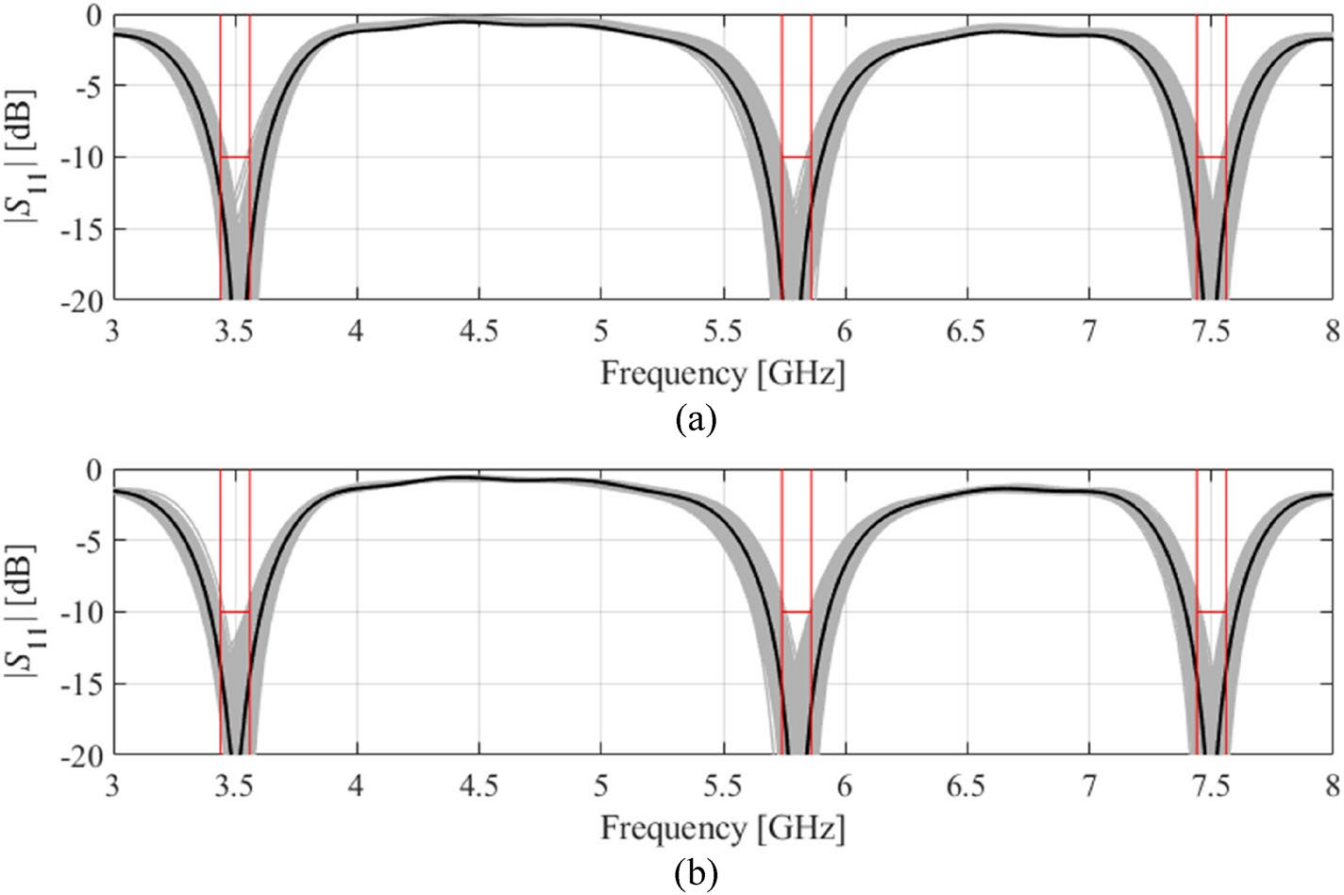
Visualization of EM-driven Monte Carlo simulation of Antenna IV using 500 samples. Gray plots represent random observables, whereas the black plot is the antenna response at the center design (nominal for (**a**) and yield-optimized for (**b**)). Target operating bandwidths and reflection level has been marked using vertical and horizonal lines, respectively: (**a**) nominal design, (b) robust design.

Table 3 shows other relevant data, including the surrogate-estimated and EM-based yield values at the nominal and optimized designs, as well as the computational cost of the optimization process for the proposed framework, and the two benchmark methods, Algorithms 1 and 2 of section “Benchmark algorithms”. Finally, Figs. 4, 5, 6 and 7 show visualization of Monte Carlo simulation for the considered antennas at the nominal and yield-optimized designs for Antennas I through IV, respectively.

**Table 3:** Yield optimization results for Antennas I through IV.

Antenna	Optimization algorithm	Initial yield		Optimized yield		CPU Cost^$^
		Estimated by surrogate model (%)	EM-based* (%)	Estimated by surrogate model (%)	EM-based* (%)	
I	Algorithm 1	81	81	92	93	400
	Algorithm 2	81	81	91	91	150^#^
	This work	80	80	91	91	72
II	Algorithm 1	64	65	95	94	800
	Algorithm 2	64	65	93	92	150^#^
	This work	64	65	91	92	60
III	Algorithm 1	63	58	75	66	1600
	Algorithm 2	62	58	72	69	400^#^
	This work	55	58	66	69	162
IV	Algorithm 1	30	36	61	54	1600
	Algorithm 2	33	36	56	59	450^#^
	This work	39	36	68	63	167

^$^Optimization cost in number of EM analyses of the antenna structure.

^#^The cost calculated as the number of algorithm iterations multiplied by the number of training samples used to set up surrogate for each iteration (50 for Antennas I and II, and 100 for Antennas III and IV).

^&^The cost includes sensitivity estimation (*n* EM analyses, cf. (7)), and acquisition of training data for surrogate model construction.

*Estimation based on Monte Carlo simulation using 500 random samples.

### Benchmarking and discussion

The numerical data provided in Table 3 demonstrates computational advantages that can be achieved by means of the proposed approach. These mainly stem from a small volume of the surrogate model domain defined with the essential directions, which is, at the same time, of sufficient extent to maximize yield without the necessity of rebuilding the metamodel.

Consequently, the average computational cost of the robust design process is as low as 115 EM analyses of the antenna structure at hand. For benchmark Algorithm 1, the cost is much higher as the surrogate is constructed in a relative large domain. In the case of benchmark Algorithm 2, the individual cost of surrogate model construction is low, however, the process of relocating the design and setting up the metamodel is iterated. The computational savings obtained using the method proposed here over Algorithm 1 are 82, 93, 90, and 89 percent (average of 88 percent) for Antennas I, II, III, and IV, respectively. The saving over Algorithm 2 are 52, 60, 60, and 63 percent (average of 59 percent).

The reliability of the optimization process is comparable for all methods in terms of the final yield value (the differences are at the level of a few percent, which is not significant). The accuracy of yield prediction is also similar for all methods, as corroborated through EM-based Monte Carlo analysis.

However, it is generally lower for Antennas III and IV, which is because the predictive power of the surrogate models is degraded as compared to Antennas I and II, due to a larger number of geometry parameters and more complex response structure (three operating bands). For example, for Antenna I, the relative RMS error of the surrogates are 0.7% (Algorithm 1), 0.4% (Algorithm 2), and 1.2% (proposed method). For Antenna II, we have 1.3% (Algorithm 1), 0.9% (Algorithm 2), and 1.4% (proposed method). Whereas, for Antenna III and IV, the respective figures are 2.8%, 2.1% and 1.6% (Algorithm 1, Algorithm 2, and the proposed method), and 7.6%, 4.0%, and 3.6% (Algorithm 1, Algorithm 2, and the proposed method), respectively. This is despite using a considerably larger number of training data samples for Antennas III and IV. For Antenna IV, the predictive power is limited as compared to other structures, which results in much larger discrepancies between the surrogate-predicted and Monte-Carlo-simulated yield values. The above data also indicates that the considered verification cases are challenging, and a construction of reliable surrogates is difficult even for relatively narrow ranges of geometry parameters.

It should be mentioned that the numerical results are not supported here by experimental validation, which is for several reasons. First, experimental estimation of statistical figures of merit (here, the yield) would require massive fabrications of antenna structures, preferably in independent manufacturing runs, which is practically infeasible. Second, the numerical experiments have not accounted for all possible uncertainties (e.g., antenna assemly inaccuracies, deviations of material parameters from their nominal values, etc.). Finally, physical measurements of the antenna prototypes are prone to errors, the level of which is typically higher than antenna response variability due to tolerances.

## Conclusion

This work introduced a novel surrogate-assisted technique for low-expedited and reliable robust design of antenna structures. The fundamental concept behind the proposed approach is to employ problem-specific knowledge to set up the surrogate model in a low-volume domain, so that the cost of training data acquisition is limited, yet the region of model validity extends sufficiently to allow yield maximization in a single step, without the necessity of rebuilding the surrogate. This is achieved by using so-called essential directions extracted in an automated decision-making process to maximize antenna response variability, with a limited number of such directions employed to span the metamodel domain. The validation results obtained for four microstrip antennas, including single-, dual-, and triple-band structures, corroborate the efficacy of the presented technique, both in terms of its low execution cost and reliability. The average cost of yield optimization is only 115 EM analyses of the antenna of interest, whereas the design quality (final yield value) is similar to the benchmark methods. At the same time, our methodology allows for significant savings over the reference surrogate-assisted methods. The speedup is as high as 88 percent on the average over the single-surrogate algorithm, and 59 percent over the sequential approximate optimization approach. Furthermore, yield estimation using the domain-restricted surrogate is at the same level of reliability as for the benchmark, as confirmed through EM-driven Monte Carlo analysis.

## Data Availability

The datasets generated during and/or analysed during the current study are available from the corresponding author on reasonable request. Contact person: anna.dabrowska@pg.edu.pl.
